# Flexible multichannel electrodes for acute recording in nonhuman primates

**DOI:** 10.1038/s41378-023-00550-y

**Published:** 2023-07-20

**Authors:** Yang Wang, Qifan Wang, Ruichen Zheng, Xinxiu Xu, Xinze Yang, Qiang Gui, Xiaowei Yang, Yijun Wang, He Cui, Weihua Pei

**Affiliations:** 1 State Key Laboratory of Integrated Optoelectronics, Institute of Semiconductors, Chinese Academy of Sciences, Beijing, 100083 China; 2 University of Chinese Academy of Sciences, Beijing, 101408 China; 3 Institute of Neuroscience, Key Laboratory of Primate Neurobiology, CAS Center for Excellence in Brain Science and Intelligence Technology, Chinese Academy of Sciences, Shanghai, 200031 China; 4 Chinese Institute for Brain Research, Beijing, 102206 China

**Keywords:** Biosensors, Electrical and electronic engineering

## Abstract

Flexible electrodes have demonstrated better biocompatibility than rigid electrodes in relieving tissue encapsulation and long-term recording. Nonhuman primates are closer to humans in their brains’ structural and functional properties, thus making them more suitable than rodents as animal models for potential clinical usage. However, the application of flexible electrodes on nonhuman primates has rarely been reported. In the present study, a flexible multichannel electrode array for nonhuman primates was developed and implemented for extracellular recording in behaving monkeys. To minimize the window of durotomy for reducing possible risks, a guide-tube-compatible implantation solution was designed to deliver the flexible electrodes through the dura into the cortex. The proposed structure for inserting flexible electrodes was characterized ex vivo and validated in vivo. Furthermore, acute recording of multichannel flexible electrodes for the primates was performed. The results showed that the flexible electrodes and implantation method used in this study meet the needs of extracellular recording in nonhuman primates. Task-related neuronal activities with a high signal-to-noise ratio of spikes demonstrated that our whole device is currently a minimally invasive and clinically viable approach for extracellular recording.

## Introduction

Compared with rigid electrodes, flexible electrodes exhibit lower bending stiffness^1^, which allows them to better accommodate micromotions of the brain^2^ and reduce mechanical interactions with surrounding tissues, thus improving the biocompatibility of the electrodes and extending the lifetime of in vivo recordings. Flexible electrodes are typically prepared by microelectromechanical system (MEMS) processes, exhibiting great potential in achieving small footprints and high throughput. Wei et al. used nanofabrication technology^3^ to reduce the cross-sectional area of flexible electrodes to less than 10 μm^2^. The neurotassel electrode^4^ reported by Guan et al. had a cross-sectional area as low as 3 × 1.5 μm^2^. Yang et al. proposed a neuron-like flexible electrode^5^ whose cross-sectional area could be as low as 1 × 0.9 μm^2^. These ultrasmall flexible electrodes had been demonstrated to have little chronic immune response over several months of implantation. In terms of high-throughput integration, Chuang et al. validated 1024-channel flexible electrodes based on a modular stacking design in rats^6^. Musk et al. integrated 3072-channel flexible electrodes on a custom chip and verified them in vivo^7^. This technology is promising for providing an interface between the brain and machine for human beings. However, flexible electrodes have been mostly tested on rodents. As an animal model closer to humans, nonhuman primates (NHPs) have a larger brains than rodents, which means that electrode design (length, site spacing, number of channels, etc.) and encapsulation methods need to be adapted to NHPs. Another difficulty is the vast time consumption of flexible electrode implantation tests. For instance, single surgical implantation and the full recovery period usually take several weeks for monkeys. Furthermore, each implantation could cause some loss of neurons, making the implanted area nonreusable.

To verify the in vivo recording characteristics of flexible electrodes in primate brains, a flexible multielectrode array (fMEA) with good flexibility and electrochemical properties was designed and prepared in accordance with the primate brain structure in the present study. Compared with rigid multielectrode arrays (MEAs), fMEAs can be prepared in batches with a small footprint, high density, and good uniformity. They can also meet the need for recording in different brain regions, different depths, and different densities. The common implantation of flexible electrodes requires a craniotomy and durotomy to expose the cortical tissue, avoiding the obstruction of the tough dura and allowing precise electrode positioning. However, removing the dura carries a high risk and a host of postoperative problems, such as cerebrospinal fluid (CSF) leakage, inflammation, and a severe neuroimmune response^8,9^. In particular, it is unfeasible for flexible electrodes to penetrate brain tissue by their own stiffness. Therefore, a microneedle-assisted and guide-tube-compatible implantation method was proposed to insert flexible electrodes into a macaque monkey’s dorsal premotor cortex (PMd) without removing the dura, significantly reducing the implantation injury.

## Results

### Design of the fMEA and the implantation method

**Fig. 1:**
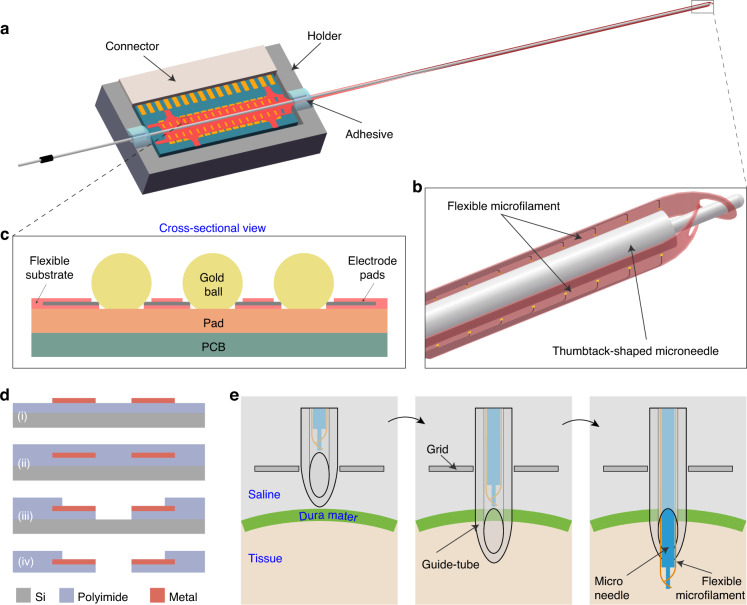
Design of the fMEA and the implantation method. **a** The 3D schematic of the assembly of flexible microfilaments and rigid microneedles. **b** Enlarged view of the tip of the "needle-filament" structure, here are two back-to-back flexible microfilaments threaded on the thumbtack-shaped microneedle. **c** Gold-ball bonding used for connecting the flexible electrodes to the PCB. **d** Process flow of flexible electrode fabrication. **e** Schematic diagram of the electrode implantation with the aid of a “needle-filament-tube” structure without removing the dura

According to the target brain region and the recording chamber depth, the fMEA was designed to be two parallel microfilaments that are 60 mm in length (Fig. 1a). There were 16 electrode sites on each microfilament, arrayed at the tip, with a total span of ~3 mm. The electrode sites had a diameter of 15 μm and a center-to-center spacing of 200 μm. There was a 110 μm × 170 μm slot hole at the front end of the microfilament (Fig. 1b). A rigid microneedle with a thumbtack-shaped tip could pass through the slot to carry the flexible microfilaments into the brain tissue. The two flexible microfilaments were threaded back-to-back on the microneedle to increase the recording channels in the implanted location. At the rear end of the microfilament, gold balls rather than wires were used to reduce the bonding area^10^ (Fig. 1c). Each electrode pad had three vias arranged at 150 μm spacing, and the diameter of the vias was 60 μm. Each via could independently connect the electrode to the printed circuit board (PCB) using gold balls with a diameter of 70–80 μm. Such a redundant design was able to prevent the vias from being damaged during the fabrication process or the gold ball from failing to bond with the electrode pad, improving the reliability of the electrode.

In typical acute implantation with a rigid electrode, the electrode punctures the dura directly or is guided by an inserted tube^11–14^. Prepuncture of the dura with a guide tube can protect the tip of the rigid electrode and support the implementation. In contrast, flexible electrodes are usually implanted with dural removal^10,15^. Here, an implantation method called “needle-filament-tube” was designed to allow the direct insertion of flexible electrodes into the brain without removing the dura. The method was based on the mutual coordination between the flexible microfilaments, the rigid microneedle, and the guide tube (Fig. 1e). A tungsten/stainless-steel microneedle was machined into a thumbtack shape, with an abrupt change in diameter between the shank and the tip of the microneedle. The tip had a diameter of ~80 μm, allowing it to pass through the slot hole at the front of the flexible microfilament, while the shank part was 220 μm in diameter, much larger than the slot hole (Fig. 1c). This design ensured that the tip of the microfilament was always aligned with the tip of the microneedle during assembly and implantation. There was no displacement when the force was applied, which helped to precisely position the electrode in depth.

### Ex vivo characterization of the fMEA

An ex vivo experiment was performed to test the reliability of the proposed structure and method for the implantation of flexible electrodes. Electrochemical methods were used to characterize the basic electrical performance of the electrodes for extracellular recording.

#### Fabrication and packaging of the fMEA

**Fig. 2:**
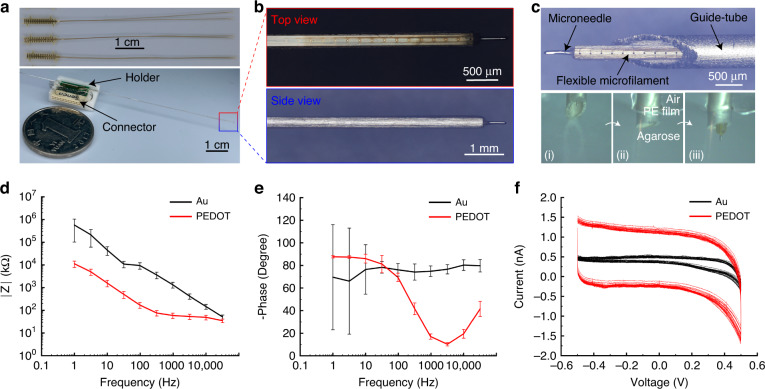
The ex vivo characterization of the fMEA. **a** 32-channel fMEA and assembled "needle-filament" structure. **b** Top and side view of the tip of the “needle-filament” structure, showing the recording sites facing outward and a diameter close to the original microneedle. **c** The “needle-filament-tube” structure (above) and the simulated implantation (below): (i) The tip of the guide-tube came into contact with the PE film; (ii) The guide-tube penetrated the PE film; (iii) The microneedle carried the flexible microfilament into the agarose. **d**–**f** The electrochemical characterization of flexible electrodes before and after modification of PEDOT (*n* = 25). **d** The frequency–impedance spectrum. **e** The frequency–phase spectrum. **f** The CV curves

As shown in Fig. 2, two 6-cm-long flexible microfilaments were assembled on a stainless-steel microneedle with a diameter of 220 μm. There was no significant increase in volume between the assembled “needle-filament” structure and the original microneedle (Fig. 2b) because the fabricated flexible microfilaments were only 6 μm in thickness. The “needle-filament” structure could move freely in a guide tube with an inner diameter of ~400 μm without any significant friction (Fig. 2c).

#### Simulated implantation in the brain phantom

We used agarose as a substitute for brain tissue and polyethylene (PE) film for dura to construct an environment simulating the mechanical properties of the primate brain for fMEA implantation. The results showed that the assembled “needle-filament-tube” structure was strong enough to allow the flexible electrode to penetrate the agarose with the aid of the microneedle after the guide tube pierced the PE membrane. During ten repeated implantations, there was no detachment, misalignment, breakage of the microfilaments, or bending of the microneedle (Fig. S1). These results demonstrated that the fabricated flexible electrodes were mechanically robust, and the proposed “needle-filament-tube” solution showed little difference from conventional rigid electrode implantation in terms of implantation strength and convenience (Fig. 2c).

#### Electrochemical characterization of the fMEA

The frequency–impedance and frequency–phase curves before modification demonstrated typical gold interface characteristics (Fig. 2d, e). The impedance value of the tested 25 electrode channels was 1.33 ± 0.38 MΩ (mean ± s.e.m.) at 1 kHz. After modification with PEDOT, the electrode impedance significantly decreased, and the average value dropped to 59.5 ± 15.6 kΩ (mean ± s.e.m.). The CV results showed that the charge storage capacity of the electrode surface modified with PEDOT was significantly increased (Fig. 2f).

### Neural recording in the behaving monkey

The ex vivo tests preliminarily revealed the basic properties of the electrodes. However, the in vivo test could reflect the performance of the electrode in complex intraorganismal environments. In the present study, an acute implantation method was developed, and neural signals were recorded from the behaving monkey by fMEA. A macaque monkey (male, 10 kg) was used for the in vivo test (Materials and methods “Behavioral task”). Spike signals were acquired in five of the seven recording sessions. These five sessions were defined as effective implantations. The spiking yield was 33 ± 11% (mean ± s.d.) across all the effective implantations.

#### Neurophysiological experiment and recording performance

**Fig. 3:**
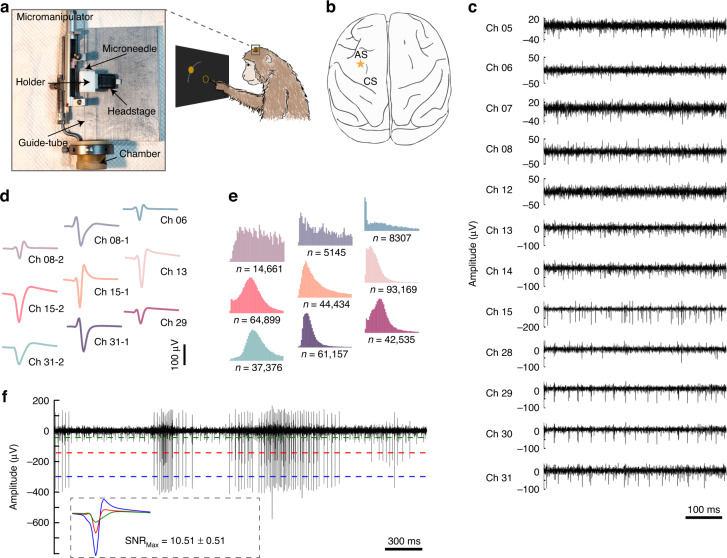
Neurophysiological experiment and recording performance. **a** Neural recording setup with a monkey doing flexible manual interception task. Left, the micromanipulator with the electrode array and a 32-channel amplifier. Right, the monkey was trained to hold center and wait for the peripheral target appearance (targets have three speeds—120°/s, 0°/s, and 120°/s), then reach out to intercept the target (Materials and methods 4.4). **b** The recorded location in PMd. **c** Neural signals recorded simultaneously in an example session of 32 channels with a time window of 500 ms. **d** The waveforms after spike sorting, some channels recorded the same unit (e.g., Ch 29 and Ch 30), the vertical bar indicates 100 μV. **e** The distributions of interspike intervals, *n* is the spike numbers of each single unit. **f** Neural signals acquired from fMEA recorded in PMd after 300–3000 Hz bandpass filtering. The left-bottom panel shows the waveforms of three units after spike sorting, the SNRs of the three units are 10.51 ± 0.51, 5.18 ± 1.66, and 1.83 ± 0.35 (mean ± s.d.), respectively

An acute recording method for NHPs was adapted for fMEA recording. The skull under the recording chamber was removed surgically, and the dura was penetrated with a guide tube during the recording session (Materials and methods “Neural and behavioral recordings”). A microneedle was used to carry the flexible electrode array into the cortex. The diameter of the perforation to the dura was ~0.3 mm^2^, which could minimize injury. Figure 3a shows the head-fixed monkey with a flexible electrode recording setup while performing the manual interception task^16^. This protocol was not dependent on anesthesia and was easily performed while the monkey was awake. In addition, it did not require additional postsurgical recovery time. The overall setup greatly facilitated experimental iterations for testing the design of electrodes. It should be noted that the shuttle device was retained during the acute recording. Figure 3b shows the location of the recording in PMd. In the example session in Fig. 3c, the spike yield is shown (12/32 channels), with some of them recording the activity of the same neuron. After preprocessing, the data were spike-sorted by WaveClus. Nine units are shown in Fig. 3d, and the distributions of the interspike intervals are shown in Fig. 3e. The quantitative measures (L_ratio_ and isolation distance^17^) of recorded units in the example session are shown in Table S1 and Fig. S2. The signal-to-noise ratios (SNR) of the spikes recorded by the PEDOT-modified electrode reached 10.51 ± 0.51 (mean ± s.d.) with a baseline of ~20 μV (Fig. 3f).

#### Example recorded neurons

**Fig. 4:**
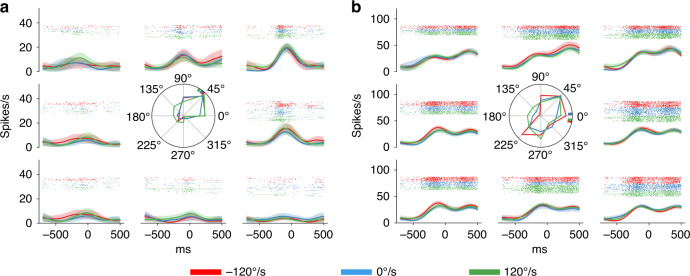
The task-relevant neurons recorded by the flexible electrodes. **a**, **b** show PSTHs of two example neurons with time aligned to the movement onset, subplots in peripheral eight directions equally dividing 360° show different neuronal activity for corresponding reach directions. The subplots in the middle indicate preferred directions (weighted mean activity, denoted by a short bar) near the movement onset (±100 ms)

Neurons in the motor cortex have been found to encode various kinetic and kinematic parameters. In particular, PMd has long been thought to be involved in the preparation and execution of hand movements^18^. In center-out reaching task studies, cortical motor neurons have shown cosine tuning for reach directions^19,20^. This characteristic is widely used in the neural decoding algorithms of brain-computer interfaces^21,22^. For the manual interception task, the condition in which the target velocity is zero is the center-out reach task (Materials and methods “Behavioral task”). Different firing patterns were observed during the perimovement period. Figure 4a, b show two example neurons’ peristimulus histograms (PSTHs) with unimodal and bimodal patterns, respectively. The recorded neuronal activity also showed a rough direction preference in cosine tuning for both static and moving targets. The results showed that the neurons recorded by the flexible electrodes could provide a wealth of task-relevant information.

## Discussion

Flexible MEAs have received much attention because of their potential for chronic recording. However, for acute recording, they still have the following advantages: (1) Compared with handmade rigid MEAs such as microwire arrays^8,23,24^, flexible MEAs can be manufactured in batches with smaller sizes, higher density, and better uniformity due to their compatibility with MEMS processes. (2) Flexible MEAs can be used for recording in deep brain regions of larger animals. For silicon-based MEAs, their shank length is usually no more than 10 mm^25,26^, which makes deep brain regions of NHPs untouchable. (3) The channel numbers of flexible MEAs can be adjusted according to the recording requirements. For example, more than two filaments can be assembled on one microneedle to acquire activities of brain regions with neurons in higher density, such as the cerebellar cortex, using the same electrode design but with no more tissue injury (Fig. S3a). (4) Flexible MEAs allow for the distribution of recording sites at different depths across different brain regions (Fig. S3b). The location and spacing between multiple target brain regions could be arbitrary. Due to the flexibility of the electrodes, the head stages at the back end can be arranged together to reduce the package volume.

Conventional implantations of flexible electrodes need to open the dura and penetrate the cortex with a shuttle device^10,15,27^. However, durotomy with an open field is accompanied by high risks, such as CSF leakage and inflammation. The proposed “needle-filament-tube” implantation method significantly reduces invasiveness. It also decreases the testing period from several weeks to days, which relieves animal suffering and enables rapid iterations of electrode designs. However, there are still some limitations in the present study. Both the guide tube and microneedle were retained during the recording. Although this is acceptable for acute recordings with head-fixed animals, it limits the application in chronic recordings. Hence, there is still a need to develop a customized implantation device to remove the guide tube and microneedle in chronic implantation surgery. By combining our acute recording method with preoperative localization, intraoperative electrophysiological recording to check the implantation depth, and a noninvasive near-infrared vein finder to avoid large vessels^28^, all the advantages mentioned above can be inherited in the development of a chronic implantation protocol. This method is significantly less invasive and can maintain the flexibility of implantation, giving it great potential in clinical applications.

For electrophysiological recordings, impedance (at 1 kHz) is an indication for evaluating the performance of electrodes. Typically, the impedance of recording electrodes for extracellular recording is in the tens to hundreds of kiloohms^10,29^. It is mostly believed that electrode impedance affects signal quality^30^, while a few studies are against it, especially during in vivo recording^28,31,32^. Reducing the surface area of the recording site benefits single-unit recording and enables miniaturization of the electrode size. However, reducing the surface area also increases the electrode impedance, which may result in higher thermal noise. A common way to reduce the impedance while maintaining the size of the electrode sites is to modify the electrode surface, such as using PEDOT. We tested gold surface and PEDOT-modified electrodes but found no significant difference in baseline noise level. The baseline noise level could be affected by many factors in vivo, where electrode thermal noise is not necessarily the dominant source of the noise together. The amplifier noise and other distant neurons’ activity contribute to the baseline noise. The magnitude of the SNR often depends on the recording location, i.e., the distance of the electrode from the recorded neuron. The golden criterion of suitable electrodes is whether task-relevant information can be efficiently and stably extracted from the recorded signals.

## Materials and methods

### Fabrication and packaging of the fMEA

The fMEA was prepared by the MEMS process with the following procedure (Fig. 1d): (1) Spin-coating liquid polyimide (PI2611) on a silicon wafer and then baking the wafer on a hot plate (60 min at 300 °C) to form the bottom PI layer. (2) Lithographing negative photoresist (AR-N-4340) to transfer the electrode patterns to the bottom PI. (3) Depositing metals (Cr/Au/Cr) and then peeling off to form the metal electrode layer. (4) Repeating step (1) to obtain the top PI layer and anneal the wafer at 350 °C. (5) Lithographing positive photoresist (AZ4620) and then exposing the electrode sites and pads by reactive ion etching (RIE). (6) Repeating step (5) to etch out the electrode profile, vias in the pads, and the slot hole at the tip. (7) Corroding the upper layer of chromium to expose the gold interface. (8) Soaking the wafer in deionized water and using a tweezer to lift up the flexible electrode at the rear end and slowly pull up the whole electrode.

Then, the microfilaments were connected to the PCB by gold-ball bonding (Fig. 1c). The gold balls were bonded to the PCB pads using a wire-bonding machine, through which the position, diameter, and bonding pressure of the gold balls could be controlled. Ultraviolet (UV) glue was applied to the bonding area for insulation and waterproofing. Finally, the PCB was soldered to a 32-channel Omnetics connector to complete the packaging of the fMEA.

### Assembly of flexible microfilaments with the rigid microneedle

The flexible microfilaments were manually threaded onto the microneedle under the microscope. During this process, microfilaments needed to be placed with their back against the surface of the microneedle to ensure that recording sites were facing outward (Fig. 1a, c). In addition, the two microfilaments needed to be placed back-to-back on two sides of the microneedle, preventing the electrode sites from being too close and enabling a more extensive recording range of neurons. After threading, the microneedle was placed parallel to the back of the PCB, and the microfilaments were stretched tightly against the surface of the microneedle. UV glue was used at the back end to fix the microfilaments and microneedles onto the PCB. Then, the assembled electrode was placed in a 3D-printed holder with a square trench and a semicircular slot reserved for its positioning. Finally, the holder and the PCB were fixed with UV glue to form a robust assembly.

### Electrochemical characterization and modification of the fMEA

An electrochemical workstation (CHI660D) was used to perform frequency–impedance spectroscopy, cyclic voltammetry (CV), and surface modification, with the microelectrodes as the working electrodes and a Pt electrode as the counter electrode. The impedance and CV were measured in PBS solution. Poly(3,4-ethylenedioxythiophene) (PEDOT) was deposited in an aqueous solution consisting of 0.02 M EDOT monomer and 0.1 M TsONa (sodium p-toluene sulfonate) electrolyte using the constant current method with a current magnitude of 6.35 nA for 30 s per electrode.

### Behavioral task

An adult male macaque monkey (*Macaca mulatta*, weighing 10 kg) participated in this study. The monkey was trained to perform a visual-guided manual interception task^16,33^, in which some task parameters were modified and briefly described here. The monkey was first required to press and hold a yellow target in the center of the screen for 400–600 ms, and then a yellow target moving in a circular path appeared once the central dot was turned off. The monkey might intercept the target by arm movement within 100–1000 ms with a tolerable error radius of 3 cm. The peripheral target moved at three angular speeds, −120°/s (counterclockwise), 0°/s (static), and 120°/s (clockwise), with equal probability of pseudorandom occurrence. The touched position after successful interception is shown in red when correct and in blue when incorrect.

### Neural and behavioral recordings

After the monkey was well trained, we performed sterile surgery, and two headposts were implanted. Anesthesia was introduced by 10 mg/kg ketamine and sustained by 2% isoflurane. A recording chamber (diameter = 19 mm, Crist Instrument) was implanted stereotaxically centered over the arm area of the premotor cortex and primary motor cortex guided by structural magnetic resonance images before the surgery. All implanting locations of fMEA were in PMd (the anterior part of the chamber). The skull under the chamber was removed, but the dura was intact. Intracortical microstimulation (Alpha Omega) was used to confirm the recorded location by evoking the muscle activity of the contralateral forelimb (biphasic pulses, 200 μs per phase, 300 Hz, cathode leading, 10–100 μA) and driving the “needle-filament-tube” structure to the target region. The guide tube and microneedle were retained during the recording. The Blackrock recording system was used with a sampling frequency of 30 kHz for the extracellular electrophysiological recordings. Visual stimulus presentation and interaction during the task were achieved via a touch screen (Elo, 60 Hz). The behavioral markers were recorded using MonkeyLogic.

### Data analysis

After the raw data were recorded, for the figures of the data recording performance, an elliptic bandpass filter of 300–3000 Hz was used to perform preprocessing by the *fix_filter* function in the open-source spike sorting software, WaveClus^34^. Single units were also isolated by WaveClus.

*The* signal-to-noise ratio (SNR) was calculated by using the following formula^35^:

$${{\mathrm{SNR}}}=\frac{{V}_{\max }-{V}_{\min }}{2{{\mathrm{RMS}}}},$$

where ${V}_{\max }$ and ${V}_{\min }$ are the maximum and minimum amplitudes of each sorted spike, respectively. RMS here is the filtered signal’s root mean square during the whole recording session.

Peristimulus histograms (PSTHs) were calculated by conditional averaging of the neuron’s firing rate aligned to movement onset and then smoothed. The preferred direction was calculated with perimovement activity within a time window of movement onset ±100 ms.

## Supplementary information

Supplementary information
