# Supplementary material for: Flexible multichannel electrodes for acute recording in nonhuman primates
Source: Microsyst Nanoeng. 2023 Jul 20;9:93. doi: 10.1038/s41378-023-00550-y (PMC10359297; doi:10.1038/s41378-023-00550-y)
Supplement: Supplementary file 1 — Supplementary information [file 41378_2023_550_MOESM1_ESM.docx]

Supplementary Information for

**Flexible multichannel electrodes for acute recording in nonhuman primates**

Yang Wang ^1,3,&^, Qifan Wang ^2,3,&^, Ruichen Zheng ^2,3^, Xinxiu Xu ^2^, Xinze Yang ^1,3^, Qiang Gui ^1^, Xiaowei Yang ^1^, Yijun Wang ^1,3,4^, He Cui ^2,3,4,^*, Weihua Pei ^1,3,^*

^&^ These authors contributed equally to this work and should be considered co-first authors.

^1^ Institute of Semiconductors, Chinese Academy of Sciences, Beijing 100083, China.

^2^ Center for Excellence in Brain Science and Intelligent Technology, Institute of Neuroscience, Chinese Academy of Sciences, Shanghai, 200031, China.

^3^ University of Chinese Academy of Sciences, Beijing 100049, China.

^4^ Chinese Institute for Brain Research, Beijing 102206, China.

Table of Contents:

Supplementary Figures 1 to 3

Supplementary Table 1


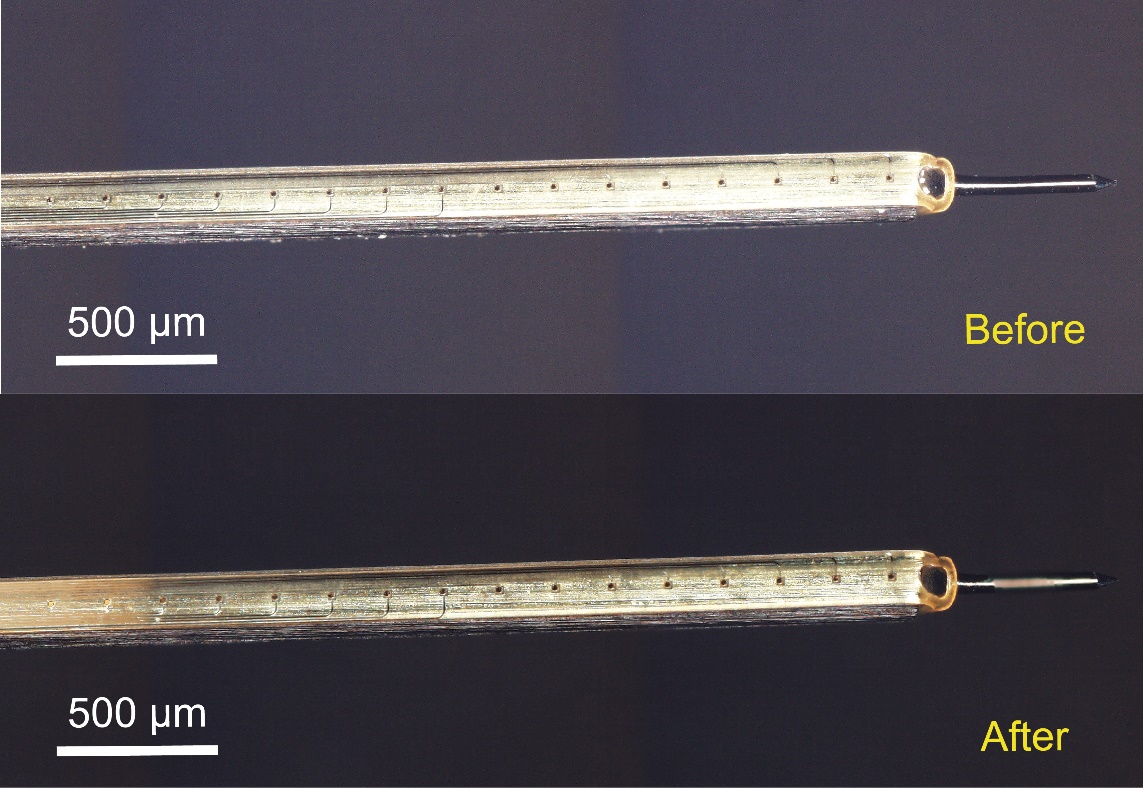


**Fig.S1** Comparison of the microneedle-microfilament structure before and after 10 repeated simulated implantations. There was neither detachment, misalignment, breakage of the microfilaments, or bending of the microneedle.

**Table S1** Quantitative measures of recorded units in the example session

| Channel | Unit | L_ratio_ | Isolation distance |
| --- | --- | --- | --- |
| 6 | 1 | 0.0410 | 36.0029 |
| 8 | 1 | 0.0605 | 25.5884 |
| 8 | ^*^2 | 0.1894 | 11.583 |
| 13 | 1 | 0.0149 | 84.6913 |
| 15 | 1 | 0.0275 | 14.5379 |
| 15 | 2 | 0.0706 | 20.5698 |
| 29 | ^*^1 | 0.1204 | 6.5502 |
| 31 | 1 | 0.0397 | 26.5492 |
| 31 | 2 | 0.0224 | 16.9400 |

* Multi-units with high L_ratio_ and low Isolation distance.

The shaded lines of Table S1 show examples of three different separation levels, good separation, bad separation, and intermediate separation, respectively (Fig.S2). The spike features (first three PCs) were used for visualization (but only the first two PCs were used to calculate L_ratio_ and Isolation distance). It should be noted that WaveClus uses the 10 most significant wavelet coefficients as features^1^.


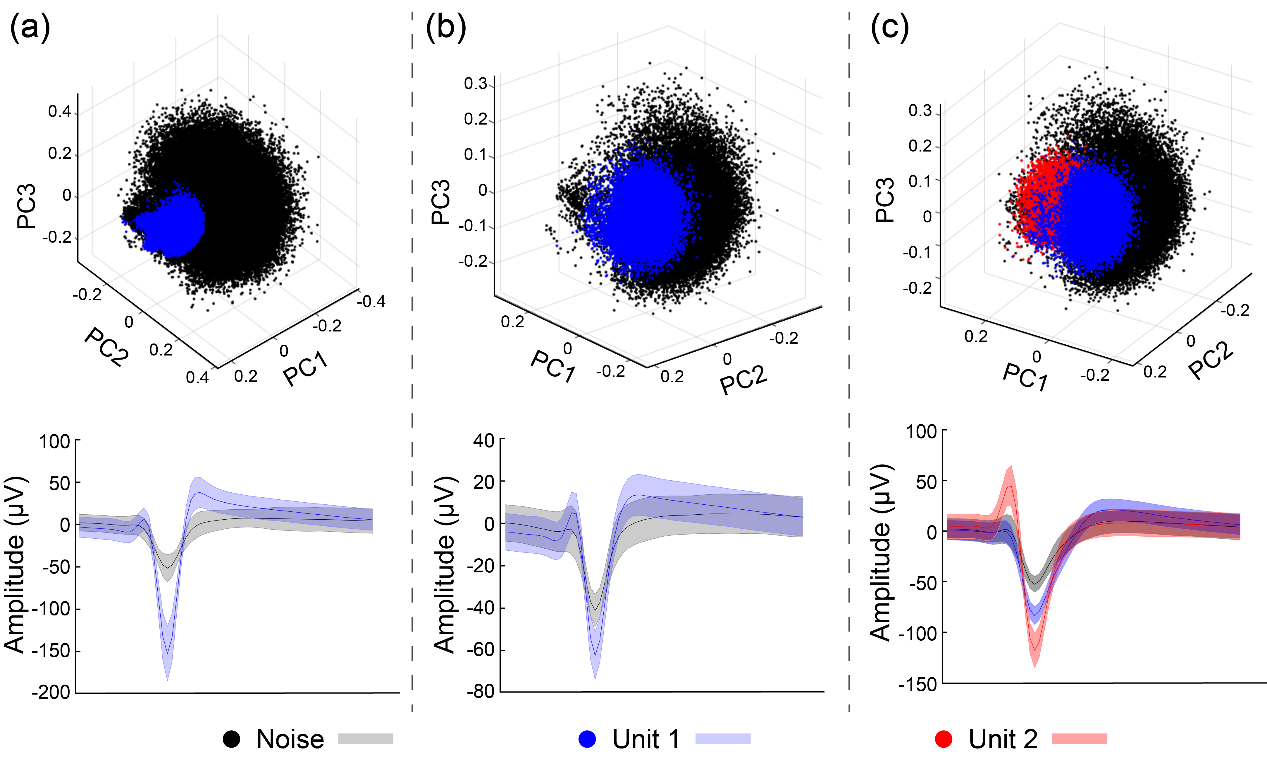


**Fig.S2** Examples of three different separation levels. (a) A good separation example: Channel 13. (b) A bad separation example: Channel 29. (c) An intermediate separation example: Channel 31. Above, the PCA visualization of clusters found by WaveClus. Below, the lines show the mean waveforms and the shaded areas show the standard deviations.


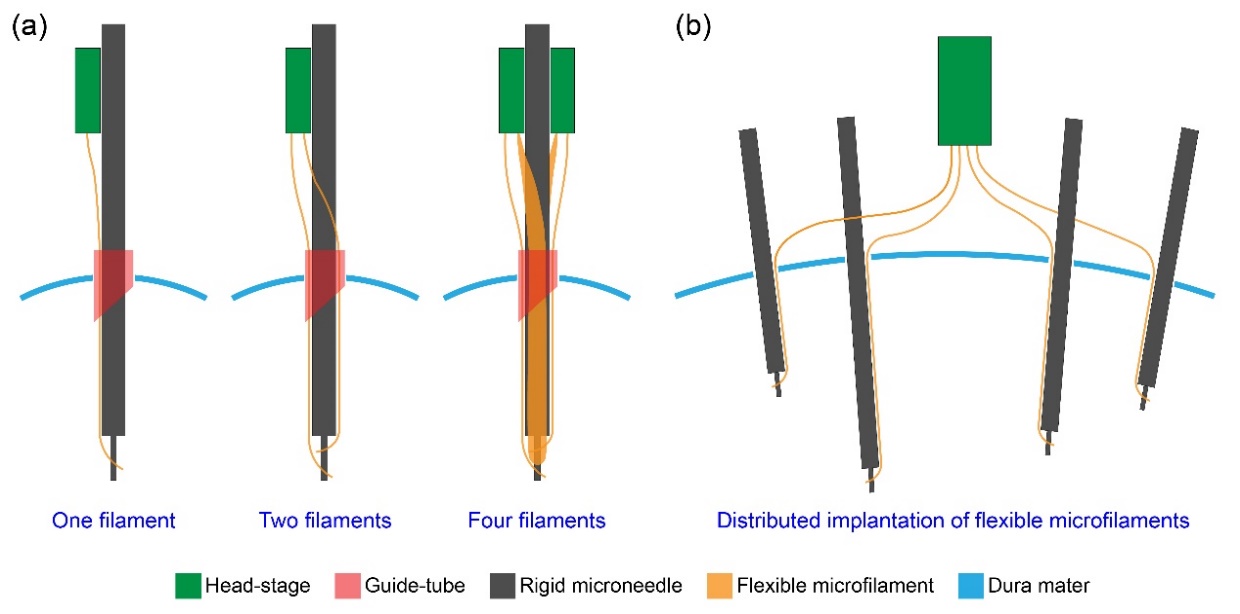


**Fig.S3** Advantages of flexible MEAs for acute recording. (a) The number of flexible microfilaments can be adjusted according to the recording requirements. (b) Flexible MEAs allow for the distribution of recording sites at different depths across different brain regions.

**Reference:**

1. Chaure F. J., Rey H. G.&Quiroga R. Q. A novel and fully automatic spike-sorting implementation with variable number of features. *J. Neurophysiol.* **120**, 1859-1871 (2018).
